# Climate change, natural resource conflicts and insecurity in Nigeria: implication for food security

**DOI:** 10.3389/fnut.2026.1805921

**Published:** 2026-05-12

**Authors:** Sarah Edore Edewor, Agatha Osivweneta Ogbe

**Affiliations:** Department of Agricultural Economics and Farm Management, Federal University of Agriculture, Abeokuta, Abeokuta, Ogun State, Nigeria

**Keywords:** climate change, disasters, farmer–herder conflict, food security, insecurity, natural resource conflicts, Nigeria

## Abstract

**Introduction:**

Climate change and rising insecurity have intensified natural resource conflicts in Nigeria, posing serious threats to agricultural productivity and household food security. This study examines the climate–conflict–food security nexus, focusing on how environmental changes contribute to conflicts and how these dynamics affect economic performance and food security outcomes.

**Methods:**

The study utilized data from the 2018/2019 General Household Survey (LSMS-ISA) and the 2022 National Agricultural Sample Census (NASC), capturing both household- and community-level information. Descriptive statistics were employed to assess patterns of climate shocks and conflicts, while econometric techniques—including Ordinary Least Squares (OLS), Ordered Logit, and Ordered Probit models—were used to analyze the drivers of food security.

**Results:**

Findings reveal that climate-related shocks and insecurity significantly increase resource-based conflicts and have strong negative effects on food security. Among the various shocks, flooding emerged as the most damaging disaster. In contrast, asset ownership was found to enhance household resilience and mitigate adverse effects.

**Discussion:**

The results highlight the need for integrated policy responses that address both environmental and security challenges. Policies promoting climate adaptation, improved natural resource governance, and conflict-sensitive interventions are essential to strengthen food security and resilience in Nigeria.

## 1 Introduction

Climate change is one of the most challenging problems facing the world in the 21st century, causing significant damages (1, 81), and a key driver of resource conflicts globally (2), particularly for regions whose economies and livelihoods are closely tied to natural resources (3, 4). This damage is more evident in sub-Saharan Africa, where agriculture remains the primary source of food, employment, and income for more than 60% of the population (5). The Africa region accounts for about 3–4% of global greenhouse gas emissions, however, countries within the region are disproportionately vulnerable to the effect of climate change basically due to its heavy reliance on rain-fed agriculture, and limited adaptive capacity (6–8).

Agriculture and pastoralism are two of the most vulnerable sectors that suffer the consequences of climate change. Agriculture highly depends on climate variables because in their appropriate levels, temperature, relative humidity, sunlight, and rainfall, are the main drivers of crop growth and yield, affecting the quantity, quality and type of food produced as well as income generated from agricultural activities. However, changes in temperature and rainfall patterns frequently increase extreme weather events like floods and drought. These changes significantly disrupt agricultural production calendars (causing uncertainty in the onset of farming season), lowering both crop yields and livestock productivity (4). Crop production suffers from both drought and flooding (waterlogging), while livestock production systems face heat stress, and reduced pasture availability. The impact is usually stronger in areas where agriculture and pastoralism play vital roles in daily subsistence and where adaptive capacity is low (81). African Development Bank (9), posit that climate change could shrink Africa’s GDP by up to 3% annually by 2030, largely due to its impact on agriculture and food systems.

Climate change increases the frequency and intensity of extreme weather events which in turn spur conflict. It also emerged as a key threat to food security especially for Nigeria which is heavily dependent on agriculture for food supply. Nigeria is largely considered for its diverse ecosystems ranging from the arid regions in the north to lush vegetation of the mangrove, rainforest, and coastal ecosystems in the south. This geographical location variation and topography largely influenced Nigeria’s climate resulting to distinct wet and dry seasons (10). This makes it vulnerable to the impact of climate change especially in the northern region, where the effect of climate change is pronounced with rising temperature, erratic rainfall pattern and increasing desertification and this has intensified scarcity of arable land and water resources (3, 11, 38). Herdsmen have been forced to migrate from the north to south due to desertification in search of pasture for their livestock and they often encroach on farmlands causing dispute with farmland owners. This often exacerbates competition over declining resources, leading to conflicts between nomadic herders and rural farmers, who both rely on the land and it resource for survival.

Apart from the impact of climate change, Nigeria has witnessed in recent times unprecedented level of insecurity challenges including herder-farmers conflicts, banditry, communal land disputes and kidnapping (12). No region in the country is spared by the insurgent of conflicts and insecurity, however, the magnitude and widespread differ across the regions in Nigeria. Among the various forms of insecurity threats, farmer-nomadic herders’ conflicts have been the most persistent. These conflicts have revolved round access to land and water resources resulting in very frequent violence (12). Agricultural production has been greatly affected leading to grave food supply crisis and deficit. The increasing occurrences of insecurity situations (killings, kidnapping and destruction of property) in many parts of Nigeria, has led to destruction of farmlands, displacement of thousands of farming households and communities, loss of agricultural labour and asset; forcing many farmers to abandon their farmland, means of livelihood and survival, to avoid losing their lives. These security threats directly undermine food production and supply with significant implications for food security, as agricultural activities are disrupted, and farmers are no longer able to cultivate and harvest their crops in sufficient and adequate quantities (13, 14).

The nexus between climate change, resource use conflict and insecurity in Nigeria can be traced to a long-standing vulnerability in agricultural production systems, where erratic weather patterns disrupt farming cycles, reducing crop yields, increases food prices; heightened competition for limited resources and threatening food security (15). Farmers and herders are crucial in providing the nutritional needs and ensuring household food security. It is often clear that there is a form of connection, several studies have investigated and analyzed this connection independently in relation to one specific issue, either as the implications of climate change (16–18) or resource use conflicts/insecurity (13, 14, 19–32, 76) on food security, rather than holistically. This study is motivated by the need to explore and quantify the implication of the nexus between climate change, resource use conflict and insecurity and household food security. The study investigates the link between climate change, resource use conflicts and insecurity and its implication for food security. Specifically, the study: (i) To assess how climate-related environmental changes contribute to resource-based conflicts; (ii) Examine natural resources conflicts and associated insecurity in relation to economic performance, agricultural productivity, and human wellbeing; (iii) Assess the implications of the climate change, natural resources conflicts and insecurity nexus on food security; and (iv) explore policy and governance responses capable of mitigating these interlinked challenges.

This study contributes to the existing literature by providing quantitative robust assessment of the implication of the nexus between climate change, resource use conflict and insecurity and household food security in Nigeria.

## 2 Literature review

Drawing the lines of causation between climate change, resource-use conflict and food security requires caution. Conflict could have many causes, and clashes usually do not ensue when the weather is heated up, or drought and desertification outcomes are experienced. For population, sectors and regions experiencing some climate shift, poor response to these shifts may lead to resource shortage and poor response to the resource shortage can intensify conflict risk.

Conflict has been a recurring decimal in the history of mankind, because of competition for scarce resources in society (22). The upsurge of conflicts (farmers-herders’ conflicts and insecurity) have make Nigeria unsafe, and these ongoing conflicts have affected the production and supply of basic food which has negatively impacted food prices and created scarcity (33). Nigeria has experienced or is still experiencing varying dimensions of conflict depending on either: banditry, Boko Haram insurgency and/or farmers-herdsmen clashes (19). This situation has decreased the standard of living, while cost of living continue to rise, Nigerians no longer have the means of affording basic food needs. Some conflicts arise between similar resource users (between one farming community and another), others occur between different resource users (conflict between pastoralists and crop farmers or between foresters and crop farmers) (21).

**Figure 1:**
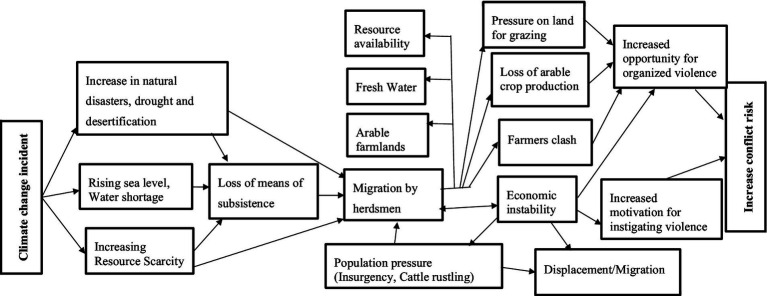
Climate change, resource use conflict and food security pathway. Source: Adapted from Odoh and Chigozie (37).

Conflict between herdsmen and farmers have since the 20th century become widespread in West Africa coastal countries (34) including Nigeria as well. The farmers-herders’ conflicts over farmland and pasture have been a continuous struggle and have escalated in many parts of the country, particularly in the northern region resulting in huge casualties and rising tensions (35). Most conflicts between herdsmen and farmers in Nigeria have become more associated with competition for natural resources (inability of herdsmen to access open grazing areas) as shown in Figure 1 (36, 37). The incident of drought, desertification and the collapsed of the Rural Grazing Area (RUGA) policy system have forced nomadic herdsmen to migrate to the middle belt and south in search of graze land for their livestock, leading to competition over scarce resources and clashes with settled farmers (35). The outcome of this is loss of lives and livelihood, disruption of food production, and in some cases, insecurity surge, mass displacement and migration of people with significant consequences for food security. Clashes between farmers-herders in Nigeria spanned decades which has resulted to various dimensions, threatening socio-economic development and food security. These clashes ensued due to farming, grazing land and water resources (39).

Fajonyomi et al. (22) posit that farmer-herdsmen conflicts have a direct impact on the lives and livelihood of the people by disrupting and threatening the sustainability of pastoral production and agriculture. In addition, not only do the conflicts have a direct impact on the lives and livelihoods of those involved, but it also disrupts and threatens the sustainability of agricultural and pastoral production and by extension, the sustainability of livelihoods of rural communities affected (40). Studies have shown that there are varying dimensions to conflict. There are farmer-farmer conflicts, pastoralists–farmer conflicts and communal conflicts (19, 21, 41). The major causes of resource-use conflict are overgrazing farmlands, increasing population and breakdown of moral economy. This reduces crop production, income and in some extreme cases rape/abduction of women and, loss of lives.

Umar et al. (13) examined the effect of insecurity on food security status of farmers in Niger State, Nigeria. The study observed that farmland destruction through insecurity had significant negative effect on food security status of the farmers household stating insurgency has negative outcome on food abundance (availability) of farmers. Asanebi (33) in the study on farmers-herders’ conflicts and food insecurity in Nigeria, posit that farmers-herdsmen conflict has an alarming impact on Nigeria’s economy, thus reducing the supply and production of staple foods, increasing food insecurity, as well as raising food prices and scarcity. It was also pointed out that land issues accounted for the highest percents of conflict, followed by struggles for water, political issues and climate change. Apart from the attended food security challenges experienced during farmers’-herders’ conflicts, there is also the ripple effect of health status of displaced farmers. Chikezie et al. (21) in assessing displaced farmers’ perception on resource use conflicts as an obstacle to household food security and food safety, observed that resource-use conflict reduced farm investment, disrupts production processes, creates food shortage, and destroys food stocks thereby increasing the level of food insecurity. They stated that in the event of conflict, food items are easily contaminated due to poor handling which leads to outbreak of food borne illness and recommend that adequate food and water supply be made available with the provision of proper health facilities to handle cases of illness outbreak.

Bassey and Ukpong (42) observed that crisis between farmers and herdsmen is one of the major issues affecting food sustainability in Nigeria, stating that the conflict has ripple effects on farmer communities, resource use, destruction of crops and livestock, displacement of persons, destruction of lives and properties and increase in prices of commodity. It suggests that government adopt a holistic approach in the management of this conflict to achieve a required outcome of security. Akinrinde et al. (43) opined that one of the quests to finding solution to the negative effect on Nigeria’s food security caused by farmer-herdsmen conflict is through the use of information and communication technology (ICT). They suggested an official establishment of a digital database for all herdsmen, crime reporting and emergency call applications and software for both farmers and herdsmen as visible mechanism to proffering solution to the lingering farmer-herdsmen conflicts.

Persistent clashes between farmers and herders in Nigeria have continued to threaten food security. The produce of smallholder farmers and pastoralists has remained the primary source of food for many Nigerians. However, the conflict between them is threatening food security and the economy of the country (44). The implications of this conflict in communities are such that economic activities inclusive of food production are threatened thus driving widespread hunger and malnutrition (45). Food security is a situation that exist when all people, at all times, have physical and economic access to sufficient, safe and nutritious food to meet their dietary need and food preferences for an active and healthy life (46). Four key pillars are identified by this definition: (i) physical availability of food (production and supply of food); (ii) economic access of food (dependent on income and price of food on the market); (iii) appropriate utilisation (consumption and nutritional benefit gained from eating food); and (iv) stability (ability of the people to have access to an adequate food supply at a relatively stable, and affordable price). The physical availability of food is necessary for food security; however, it is not a sufficient condition for households or individuals’ food security.

Food security ensures a widespread improvement in household and individual wellbeing and/or welfare. The produce of smallholder farmers and pastoralists has remained the primary source of food for many Nigerians (42). FAO (47) reported that an estimated 7.1 million Nigerians are food insecure calling for urgent intervention on the continued unabated herdsmen-farmers conflicts and insecurity situation in Nigeria which has led to lots of farmlands been destroyed, threatening food security, and the achievement of Goal 2 of the Sustainable Development Goals (SDGs) of 2030. There has been a decline in the food security level of Nigeria since the oil price decline of 2015 coupled for with the Naria devaluation and restriction of foreign exchange on import. This has spiked an increase in household food insecurity between 2015 and 2018 by 63.2%. Households attempted to cope by engaging in agricultural activity (48) which is also frequently challenged by climate change, conflicts and insurgence.

Nigeria is currently faced by the twin problem of hunger and malnutrition due to the disruption in agricultural production activities. Extreme climate change conditions, unabated conflicts and insurgency have affected food production activities. Agricultural production has been the main stay of the country employing 70% of labour, providing a source of livelihood for farmers, and producing and supplying food for household consumption. This sector is very sensitive to climate change impacts (rising temperature, rainfall variability; late onset of rains and early cessation of rainfall, flood disaster, reduced river flow, declining water table), conflicts (farmers-herders crisis) and insurgency (49). The outcome of these extreme events (climate change, conflicts and insecurity/insurgency) are not favourable and lead to food shortage, loss of crops in the farm, loss of livelihood, reduction available natural resource (pasture for livestock feed) and increase food prices. The reduction of pasture for livestock feed and water in the norther region caused by extreme climate events (desert encroachment and drought), push herdsmen to migrate drifting southward outside their normal grazing route. This affect physical and economic access of food as production activities reduces and livelihood are lost. This triggers food insecurity in areas that are vulnerable to these events.

Several studies (Table 1) have investigated and analyzed this connection in relation to one specific issue, either as the implications of climate change on food security; or resource use conflicts and/or insecurity on food security. There is, however, gap in knowledge and dearth of literature on the implication of the nexus of climate change, resource use conflict and insecurity on food security in Nigeria. This study contributes to the existing literature by providing empirical evidence of the implication of the nexus between climate change, resource use conflict and insecurity and household food security in Nigeria.

**Table 1:** Literature gap on the nexus of climate change, resource use conflict, and insecurity on food security.

Causes	Articles	Description	Author(s)
Climate change	Effect of climate change on food security in Nigeria	The research posed that there is a well understood conceptual links between climate change and food stability and utilization, however, less is known about the quantitative impacts.	(16)
Shortage of pasture	Farmers-Herdsmen conflicts and food security in North central Geo-political zone of Nigeria.	Farmer-herdsmen conflicts have a direct impact on the lives and livelihoods of those involved, but it also disrupts and threatens the sustainability of agricultural and pastoral production and the sustainability of livelihoods of rural communities affected	(22)
Natural resource management	Natural Resource Management, Food Security and Violent Conflicts in Nigeria: Concepts, Issues and Policy Considerations	This research is a review aimed at highlighting the linkages between natural resources management, on the one hand, and food security and conflicts on the other hand. it dived into the effects of the struggle for control over natural resources and food insecurity status on conflicts in Nigeria, and the political, social, and demographic factors that may aggravate these conflicts. It suggests that government should be attentive to the nexus between natural resources and conflict by considering issues such as grievances arising from land expropriation, environmental hazards, and social disruption accompanying labour migration and perceived injustice in the distribution of resource rents. This will ensure appropriate policy discussions and measures that could be used to manage natural resources and ensure food security which may reduce violent conflict	(20)
Resource-use conflicts	Displaced farmers perception of resource-use conflicts as an obstacle to household food security and food safety in Abia State, Nigeria	It revealed that conflicts ensuing from resource use create food shortage, destroy food stocks, disrupt production processes, reduced farm investment, cause scarcity and total crop failure. It also placed burden on available resource (food aid, water resources) in displaced camp/settlement.	(21)
Shortage of pasture	Resource scarcity and farmer-herder conflict in Nigeria: implications on food security in Benue State, 2013–2019	The study interrogated the incessant farmer-herder conflicts and its implications on food security in Benue State, Nigeria. Following the Resource Access Theory (RAT) applied to this study, it revealed that resource conflicts among herders and Benue farmers is mostly due to access to, use of, and control of ecological resources such as land and water resources for the purpose of crop farming and cattle grazing. The study revealed that a significant relationship exists between resource conflict and food. This conflict threatens three of the pillars of food security (particularly, availability of food, accessibility of food, and sustainability of food supply) in Benue state.	(30)
Herdsmen/Farmers Clashes	The Impact of Herdsmen/Farmers Clashes on Food Security in Nigeria	The study found that farmers/herders’ crises have led to the displacement of farmers, destruction of lives and properties (livestock and farmland assets), owing largely to the activities of the warring parties which has made the regions unfit for habitation.	(27)
Shortage of pasture	Resource conflict and food security: implications for peace-building in Nigeria	This study investigated resource conflict and food security in Nigeria and found that there is a significant relationship between farmer-herder conflict and food security, particularly in terms of availability of food, accessibility of food and sustainability of food supply in Nigeria	(29)
Herdsmen/Farmers Clashes	Farmer-herder conflicts and food insecurity: Evidence from rural Nigeria	This examined the impacts of the incidence and severity of farmer-herder resource use conflicts on food insecurity of rural households in Nigeria. It found that both the incidence and the severity of farmer-herder conflicts increase food insecurity and increase the number of days households have limited varieties of food eaten. However, severity of farmer-herder conflict has a greater impact on food insecurity compared to incidence of farmer-herder conflict	(24)
Insecurity insurgence	Conflict-Induced Shocks and Household Food Security in Nigeria	This research provided a review and an empirical approach in assessing the conflicts and food security in Nigeria. It observed that violent conflict ensued because of competition or access to productive resource, economic inequality and religious tension. Three conflicts (Boko Haram insurgency, herder–farmer conflicts, and armed banditry) situations were examined to affect livelihood and food security of households in Nigeria. It revealed that conflict-induced shocks significantly reduce household dietary diversity and exacerbate the severity of household food insecurity. This is due mainly to resource use competition and forced migration.	(25)
Environmental/ resource conflict	Food Security, Environmental Conflicts, and the Attainment of the United Nations Sustainable Development Goals in Nigeria	Environmental conflicts have far-reaching consequences that affect food security. Food (in)security situation is worsened by disruption of livelihoods caused by displacement and migration brought by resource shortages and climate-related pressures, tensions. Frequency of land and resource competition and disputes exacerbate food insecurity	(23)
Herdsmen/Farmers Clashes	Farmers-herders crisis and food insecurity in Nigeria	The study observed that farmers-herders crisis negatively influenced the key pillars of food security (food availability, access and utilization and stability). It identified that farmers-herders clash result as a function of climate change (drought and desertification soil degradation); growing population size, global terrorism, crime (rural banditry and cattle rustling). These crises have led to displacement of people from their locality, made women and girls’ vulnerability to sexual assault and economic predation.	(28)
Insecurity factors	Effect of insecurity on food security status of farmers in Niger state, Nigeria	It posits that frequency of attack, kidnapping, farms destroyed, were among the factors influencing food insecurity of the farmers. It suggested that insecurity activities were perceived not to have effect on food safety.	(13)
Extreme weather events land, and resource scarcity	How climate change induced land conflicts and food insecurity in Africa. A case of herdsmen-farmers crisis in Nigeria	The research highlights the adverse impact of climate change variables (extreme weather events, rising temperature and changing rainfall pattern) on agricultural practices and resource competition. It provided insight on how climate change has contributed to land conflicts and food insecurity in Nigeria.	(75)
Extreme weather events	The Effects of Climate Change on Food Security in Nigeria: A Review	This study revealed that there is a critical relationship between climate change and food insecurity, affirming that increasing frequency of extreme weather events (, rising temperatures, and changes in rainfall patterns) poses significant threats to agricultural productivity and food security in Nigeria by severely impact crop yields, disrupt food supply chains.	(17)
Extreme weather events	Effect of climate change on food security and sustainable development in north-east geo-political zone, Nigeria	This research investigated the *effect climate change has on food security and sustainable development in the zone. It revealed that* climate change is threat to food security and sustainable development in North east, Nigeria. It necessitates stunted growth in plants, poor harvest, causes land degradation, drought and desertification	(18)
Herdsmen/Farmers Clashes	Violent herder–farmer conflicts and human security in Nigeria: a focus on food security	The article observed that conflict threatens human security, especially food security, identifying that scarce land for farming and grazing results in aggressive response leading to human displacement, destruction of lives, farmland, crops and cattle rustling, which have increased food insecurity implications. It suggests that herder migration could be reduced by establishing sustainable irrigation systems and cultivation improved pastures for grazing.	(76)
Insurgence	Conflict and Food Security in Ebonyi State, Nigeria, 2015–2023	The study examined conflicts (inter and intra state) impact on food security. Food security was affected by conflicts through disruption of food distribution channels, water reticulation, killing of farmers and destruction of farm-lands. The economic activities including agriculture in the conflict area dwindled, reduce physical access of food	(32)
Extreme insecurity issues	Impact of Insecurity on Food Security in Benue State, Nigeria	It confirmed that the insecurity situation in Benue State have disrupted agricultural activities, forced farmers to abandon farmlands and limited access to markets due to fear of road blockades and loss of lives. It highlighted the frequency and intensity of herder-farmers attacks makes it the most prevalent and recurrent form of violent which has affected food availability and access forcing many households into adopting negative coping strategies (meal skipping, dietary compromise, and reliance on food aid).	(14)
Extreme weather events, disruption in food production and clashes	Climate change, food insecurity and conflict	This examines how climate change, food insecurity and violent conflict interact. It outlines evidence on the climate-food-conflict nexus showing how climate shocks (floods, droughts, heatwaves) disrupt food production, drive up prices and erode livelihood, especially in fragile and conflict-affected settings.	(82)
Climate change variable	Climate change on food security in Africa: Meta-analysis	Using meta-analysis and systematic review, the study illustrated the complex connection that exist between climate change and food security in Africa countries. Assessing the effect of climatic variables, observed that precipitation has significant (positive) effect on food security.	(77)
Resource scarcity and clashes	Environmental resource conflicts and food insecurity in rural southeast Nigeria: implications for humanitarian and sustainable development policies.	The resources-access crisis and climate change issues have affected the four pillars of food security, through encroachment into the farmland by herdsmen. It has also resulted in the destabilization of farm settlements and insecurity among communities	(31)
Extreme weather events, and resource competition	Climate change, resource conflict in Nigeria, and the immigration problem for the rest of the world	The research highlights the impact of climate change-induced resource conflicts in Nigeria, due to worsening situation of desertification and environmental degradation. This has led to clashes between nomadic herdsmen and sedentary farmers, driven by competition for increasingly scarce resources such as land and water. It has severely disrupted agricultural productivity in Nigeria’s food basket, contributing to food inflation, scarcity, and an overall economic depression. Consequently, Nigeria has experienced substantial surge internal displacement and a in international migration.	(3)
Extreme weather events, and resource competition	Reviewing the links between climate change and resource conflict	The study in it review, posits that there is an agreement that climate change influence resource conflicts. However, climate change cannot cause resource conflict in isolation, except through influence on other factors (such as land use, and population distribution) that affect resource availability, accessibility and utility. These factors are also influenced by policies and socio-cultural system. As a result, resource conflict is sometime viewed as a secondary or tertiary effect of climate change.	(78)

Source: Compiled from different sources.

## 3 Methodology

The study made use of secondary data on climate shocks, conflict and disasters and food security obtained from various sources. This includes the 2022 National Agricultural Sample Census (NASC) for Nigeria (50 × 2030 Initiative) and the 4th nationally representative Nigeria’s General Household Survey for 2018/2019 as part of the World Bank Living Standards Measurement Study-Integrated Surveys on Agriculture (LSMS-ISA) project collected by the National Bureau of Statistics (NBS). The NASC survey was conducted in 2022 to provide critical data on Nigerian agriculture. The data covers over 40 million agricultural households across Nigeria and it comprises of two components, namely the sample survey and the census (listing) components. The census component of the NASC covers information on household level agricultural activities and serves as a framework for the agricultural survey.

The NASC community data was used because it contains community data on various conflict types and natural disasters that have occurred since 2016, their economic, physical and economic impacts coupled with the severity. The LSMS-ISA data has total of five waves collected at different period with almost the same 5,000 households visited during each wave. The LSMS-ISA data collection was conducted twice for each wave: the first-round between August and October, and the second round between February and April. This data gives a more comprehensive household data on socio-demographic characteristics, land tenure, production, group leadership, food and non-food consumption expenditures, food security and shocks, and insecurity amongst others. The specific objectives were examined using the NASC (community information on disasters and conflicts) and LSMS-ISA data (which contains detailed household and individual information).

Other information was drawn from peer-reviewed journal articles and relevant reports on climate change, natural resource conflicts, insecurity, and food security in Nigeria. The analysis is guided by a climate–conflict–food security framework, which conceptualizes climate change as a structural stressor that may likely exacerbate resource scarcity, increase conflict risk, and generate insecurity that disrupts food systems.

**Figure 2:**
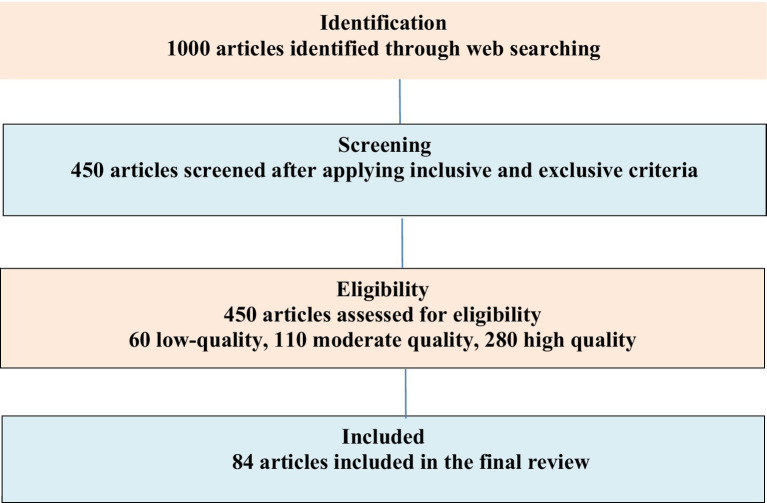
PRISMA flow diagram for systematic review.

The review of papers followed the Preferred Reporting Items for Systematic Reviews and Meta-Analyses (PRISMA) guidelines (50). A comprehensive and thorough approach was used to identify, evaluate and synthesize literature used for this study (Figure 2). The methodology encompassed an exhaustive web search strategy, rigorous quality assessment, and clearly defined inclusion and exclusion criteria. To identify relevant articles, an extensive search was conducted on Google (Google Chrome and Google Scholar). We then identified publications that mentioned the key themes in the title, the abstract, the keywords and the main text. For the identification of relevant literature, we combined keywords such as “climate change,” “farmers-herders conflict,” “resource use conflict,” “insecurity,” “food insecurity,” “food security,” “crisis,” “Migration of herdsmen” “Sub-Saharan Africa” and “Nigeria” the search. This initial search yielded 1,000 articles, papers, and documents. The selection of papers was guided by predefined criteria which include studies that focused on climate change effect, farmers-herders’ conflicts, and resource use conflicts implication on food security in Nigeria published in peer-reviewed journals, published within the specified timeframe. Excluded were studies not cantered on Nigeria, sub-Saharan Africa, and articles published before the timeframe. This filtering process reduced the pool to 450 eligible studies. Of the 450 articles reviewed, 60 were rated as low-quality, 110 as moderate-quality, and 280 as high-quality. After further refinement, only 84 articles were ultimately included in the final review.

### 3.1 Analytical technique

Data was analysed using descriptive statistics and inferential statistics. Objectives 1 and 2 were analysed using descriptive statistics while objective three was analysed using Ordered Logistic regression (Ologit), Ordered Probit regression (Oprobit) and Ordinary Least Square (OLS) regression. To measure household food security, we made use of the household food insecurity access score (HFIAS) from the GHS data.

#### 3.1.1 Household Food Insecurity Access Scale (HFIAS)

Food insecurity was measured using the Household Food Insecurity Access Scale (HFIAS) indicator. Responses were aggregated for nine standardized questions with each question capturing the frequency of food access constraints, ranging from 0 (never) to 3 (often) scores. The scores for all nine questions are summed to generate a composite index ranging from 0 to 27 for each household, with higher scores indicating greater levels of food insecurity. Based on this index, households are subsequently classified into four categories: food secure, mildly food insecure, moderately food insecure, and severely food insecure, depending on the most severe condition experienced (51).

#### 3.1.2 Ordered probit regression model

We used a range of econometric techniques to assess the effects of climate change, climate-related shocks, insecurity, and other relevant covariates on food security outcomes in Nigeria. Specifically, the Ordinary Least Squares (OLS) method is employed to estimate the relationship between the key explanatory variables and food security. In addition, both the Ordered Probit Model are utilized to estimate the probability that a household falls into a particular food security category, namely “severely insecure,” “mildly insecure,” “moderately insecure,” or “secure.”

The analytical framework for our study is anchored in the microeconomic theory of household welfare under constraints related to resources and food access and is operationalized through an Oprobit specification. Within this framework, food security is treated as a latent, unobservable construct that captures a household’s ability to consistently access adequate, safe, and nutritious food (52). However, in household survey datasets such as the Nigerian Living Standards Measurement Survey (LSMS), food security is typically observed as an ordinal variable derived from indicators like the Food Consumption Score (FCS) or the Household Food Insecurity Access Scale (HFIAS), which classify households into categories such as severely food insecure, moderately food insecure, mildly food insecure, and food secure (51). Let $y_{i}^{*}$ denote the unobserved latent index which represents the underlying level of household food security for household *i*. Following McKelvey and Zavoina (53), this index is expressed as a linear function of observable characteristics with an idiosyncratic disturbance term in Equation 1:


$$y_{i}^{*}=X_{i}^{\prime }\beta +\varepsilon_{i}$$ (1)

Where $X_{i}$ is a vector of observable characteristics capturing socioeconomic, demographic, institutional, and agroecological factors, *β* is a vector of parameters to be estimated, and $\varepsilon_{i}$ ∼N(0,1) is a normally distributed error term representing unobserved heterogeneity. We control for household demographics, economic factors (asset value, income, remittances), and location characteristics (rural/urban, region) to isolate the effects of natural disasters, climate change, insecurity, on food security. The observed ordinal outcome variable which reflects the household’s food security status is linked to the latent variable through a set of estimated threshold parameters $\mu_{j}$ such that in Equation 2:


$$=\{\begin{array}{c} \begin{array}{c} 0\;\text{if}\;\;y_{i}^{*}\leq \mu_{2}\;\;\;(\text{severely insecure})\;\;\\ 1\;\text{if}\;\mu 1<y_{i}^{*}\leq \mu_{2}\;(\text{Mildly insecure})\;\;\;\;\\ 2\;\text{if}\;\mu 1<y_{i}^{*}\leq \mu_{2}\;\mu 3\;(\text{Moderately insecure}) \end{array}\\ 3\;\text{if}\;y_{i}^{*}>\mu_{2}\;(\text{secure}) \end{array}$$ (2)

Denotes the cumulative distribution function of the standard normal distribution. The probabilities associated with each outcome are derived using the cumulative distribution function of the standard normal distribution. This formulation corresponds to the ordered probit model, which assumes the existence of a continuous latent process underlying the observed ordinal responses (54). The ordered probit specification is well-suited for modeling food security outcomes because the dependent variable is ordinal rather than cardinal.

Consistent with existing empirical studies on household food security in developing countries [e.g., (55, 56)], the explanatory variables includes both household-level and structural determinants. These include education, farm size, distance to markets, and exposure to climatic shocks amongst others. Collectively, these variables reflect both the entitlement framework (57) and the capability approach (58), which jointly shape household access to and utilization of food (Table 2).

**Table 2:** Variable description and definition.

Variable	Description
YY	Food security
Climate_exposure	Experienced at least one climate shock out of (Destruction of harvest from flood or fire, Poor rains, flooding, Pest invasion, Loss of property from flood or fire, loss of land, death of livestock and any other disaster)
Insecurity_ exposure	Experienced at least one form of insecurity (Theft of crops, Dwelling damaged and Kidnapping/Hijack)
Temperature	Average annual temperature
Rainfall	Average annual rainfall
Control variables	
Sex	Female = 1, Male = 0
Household size	Number of persons
Age	Years
Asset	Value of household asset in naira
Education	Educational Level in years of schooling
Credit access	Yes = 1, No = 0
Ext	Access to extension services, yes = 1, No = 0
Sector	Location (rural = 1, urban = 0)
Total income	All income
Farm size	Hectares
Geopolitical zones	GPZ dummies for the 6 zones (North Central, North East, North West, South East, South South, and South West)

Overall, the Ordered Probit model offers a theoretically consistent and empirically tractable approach for assessing how these factors influence the likelihood that a household attains a given level of food security. In the empirical analysis, marginal effects are subsequently computed to evaluate how variations in each explanatory variable affect the probability of transitioning across food security categories, holding other factors constant. The ordered logit model adapted for this study is linearly given as in Equation 3:


$$\begin{array}{l} YY_{i}=\alpha_{t}+\beta_{1}\text{clim}\_\exp_{i}+\beta_{2}\text{insecurity}\_\exp_{i}+\beta_{3}\mathrm{tem}p_{i}\\ +\beta_{4}\text{rainfal}l_{i}+\beta_{5}\mathrm{Se}x_{i}+\beta_{6}\text{HHsiz}e_{i}+\beta_{7}\mathrm{Ag}e_{i}+\beta_{8}\text{asset}s_{i}\\ +\beta_{9}\mathrm{Edu}c_{i}+\beta_{10}\text{credi}t_{i}+\beta_{11}\mathrm{ex}t_{i}+\beta_{12}\text{secto}r_{i}+\beta_{13}\mathrm{ex}t_{i}\\ +\beta_{14}\text{incom}e_{i}+\beta_{15}\text{farmsi}z_{i}+\beta_{16-21}\mathrm{gpz}+\varepsilon_{i} \end{array}$$ (3)

## 4 Results and discussion

This section presents the results of this study based on the study objectives.

### 4.1 Summary statistics

Table 3 presents the summary statistics of the variables used in the analysis, including the distribution of households across the food security categories. The results indicate noteworthy variations in socioeconomic and environmental characteristics of households. The average household size is 5 persons, suggesting moderately sized households, while the mean age of household heads is 49.8 years, indicating a relatively mature farming population. Majority (81%) are male and the average educational attainment was 8.63 years, implying that most had a basic to secondary-level education. A typical household had an average income of ₦343,031.90. Although the large standard deviation suggests substantial income inequality among households. Similarly, asset values show wide distribution, indicating differences in wealth status.

**Table 3:** Summary statistics of variables.

Variables	Obs	Mean	Std. Dev.	Min	Max
Household size	4,980	4.75	2.89	1	29
Asset value (naira)	5,050	175,460	437,323.5	0	1.51E+07
Farm size	3,582	1.53	8.34	0	480.17
Sex	4,965	0.194	0.396	0	1
Age	4,658	49.81	15.37	9	130
Educational level (years)	4,980	8.63	4.56	0	12
Household income	5,054	343,031.9	1,367,564	−7,601,992	4.03E+07
Credit access	4,979	0.041	0.200	0	1
Av. annual temperature	4,965	263.06	9.29	216	286
Av. annual total rainfall	4,965	1,215.83	374.92	412	2,300
Food security categories					
Severely Insecure	4,965	0.61	0.49	0	1
Mildly insecure	4,965	0.09	0.29	0	1
Moderately insecure	4,965	0.01	0.11	0	1
Food secure	4,965	0.29	0.45	0	1

Source: GHS (2018/2019).

Focusing on food security status, about 61% of households are severely food insecure, while 9% are mildly food insecure and only 1% are moderately food insecure. In contrast, 29% of households are food secure. This distribution has shown a high prevalence of food insecurity, with the majority of households falling into the severely insecure category. This suggests that vulnerability to shocks such as climate variability and conflict remains a critical issue affecting livelihoods.

### 4.2 Contribution of climate change to natural resource–based conflicts in Nigeria

Table 4 presents the structural drivers of conflicts. It highlights resource competition as the central driver of recorded conflicts. Land disputes (27.05%), livestock-related issues (24.43%), and boundary disputes (24.04%) collectively account for over 75% of conflicts with identified causes. This underscores the material basis of much Nigerian violence, rooted in competition over agrarian and pastoral resources, exacerbated by demographic pressure, climate variability, and weak land governance (59). Natural resources particularly land and water are central to Nigeria’s agrarian and pastoral economies. Approximately 70% of rural households depend directly on agriculture or livestock production (60). Farmers rely on arable land and predictable rainfall, while pastoralists depend on access to grazing land and water points. As livelihoods become less viable, affected groups are often forced to migrate in search of alternative resources, bringing them into closer contact and sometimes confrontation with other resource users. The category “Others (specify)” is minimal (3.98%), suggesting that most conflicts are attributable to these structural resource tensions rather than ideological factors.

**Table 4:** Causes of natural resource conflict in Nigeria.

Conflict cause	Percent
Land	27.05
Water	10.49
Other natural resources	10.02
Livestock related	24.43
Boundary disputes	24.04
Others (specify)	3.98
Total	100

Source: NASC (79).

This is further evident in Table 5 as it differentiates between frequent and occasional conflicts by cause. Livestock-related conflicts have the highest count of frequent instances (32.2%), indicating their persistent, recurrent nature tied to seasonal transhumance patterns and entrenched farmer-herder grievances (61). Land conflicts, while most numerous overall, are more frequently classified as occasional (22.7%), which may reflect their linkage to specific, discrete incidents of land appropriation or boundary encroachment rather than continuous tension. This aligns with the findings of Duyile and Gowon-Adelabu (62) found that climate-driven constraints on water and fodder precipitate violent clashes over resources in both Northeast and North Central states, where environmental scarcity interacts with weak governance to exacerbate tensions.

**Table 5:** Degree of conflict cause in Nigeria.

Conflict cause	Frequent	Occasional	Total
Land	728	3,218	3,946
Water	414	1,116	1,530
Other natural resources	247	1,215	1,462
Livestock related	1,036	2,528	3,564
Boundary disputes	566	2,941	3,507
Others (specify)	223	358	581
Total	3,214	11,376	14,590

Source: NASC (79).

The intensification of natural resource–based conflicts in Nigeria over the past decades represents one of the most significant internal security challenges confronting the state, with localized disputes evolving into complex security challenges with national and regional implications. Conflicts over land, water, and pasture are not new to the Nigeria context, their frequency, intensity, geographic dispersion and lethality have however, increased significantly since the early 2000s, coinciding with worsening climate variability and institutional fragility (62). Table 6 presents the degree of disaster exposure and differential vulnerability by state in Nigeria. The results demonstrate significant inter-state variation in disaster exposure. Jigawa State recorded the highest (*n* = 584), with the state heavily impacted by floods and heavy rainfall, while the Federal Capital Territory (FCT) recorded the lowest (*n* = 13). This disparity mirrors the uneven distribution of environmental risk, with states in the Niger Delta and the Sahelian belt being disproportionately exposed to hydrological and meteorological hazards. The low count in the FCT may reflect its urban character and limited agricultural base, which reduces reported disaster impacts relative to agrarian states (20).

**Table 6:** Community disaster by state in Nigeria.

State	Drought (685)	Flood (2,808)	Heavy rainfall (1,765)	Extreme Winds (1,174)	Extreme temperatures (cold/heat) (249)	Fire disaster (191)	Other (specify) (45)	Total (6,917)
Abia	0.0	0.6	0.6	0.9	0.4	0.5	2.2	0.6
Adamawa	5.0	6.3	5.0	7.7	4.0	4.7	0.0	5.9
Akwa Ibom	3.5	4.6	5.2	7.2	2.0	3.1	13.3	5.0
Anambra	0.3	3.0	0.5	0.3	0.0	2.1	6.7	1.5
Bauchi	5.5	4.6	5.3	5.5	6.4	2.1	0.0	5.0
Bayelsa	0.0	4.6	0.7	0.9	0.4	1.6	24.4	2.4
Benue	2.5	1.9	2.7	1.3	0.8	2.1	4.4	2.1
Borno	5.5	2.4	2.2	0.7	3.6	1.6	0.0	2.4
Cross River	2.5	3.6	3.1	6.0	2.0	16.8	0.0	4.1
Delta	0.3	4.9	2.5	1.5	2.0	5.8	2.2	3.2
Ebonyi	1.2	1.5	0.8	0.8	3.6	2.6	2.2	1.3
Edo	0.1	1.4	0.8	0.8	0.4	0.0	0.0	0.9
Ekiti	2.0	0.6	1.0	2.5	1.6	3.7	0.0	1.3
Enugu	0.3	0.2	0.4	0.1	0.4	0.0	4.4	0.3
Fct	0.0	0.1	0.2	0.4	0.4	0.0	0.0	0.2
Gombe	1.5	2.1	1.9	1.4	4.8	1.0	0.0	1.9
Imo	0.0	1.4	0.6	0.8	1.2	0.0	2.2	0.9
Jigawa	9.6	7.9	9.6	8.2	4.8	7.3	8.9	8.4
Kaduna	2.3	2.9	4.5	2.3	2.0	4.7	0.0	3.2
Kano	8.8	3.0	4.6	3.6	4.4	2.6	0.0	4.1
Katsina	9.6	2.7	7.8	5.6	3.6	1.0	2.2	5.1
Kebbi	3.6	4.8	4.4	2.5	7.2	0.5	0.0	4.1
Kogi	0.4	2.7	1.0	1.0	0.8	0.0	2.2	1.6
Kwara	1.5	1.0	1.5	1.4	1.6	0.0	0.0	1.2
Lagos	0.1	2.3	2.3	0.1	0.0	0.0	0.0	1.5
Nasarawa	0.7	1.3	1.4	2.3	1.2	1.6	0.0	1.4
Niger	3.1	2.1	2.2	4.3	4.8	0.0	2.2	2.6
Ogun	1.3	1.3	0.8	0.8	1.6	0.5	0.0	1.1
Ondo	0.6	2.2	1.2	3.0	0.8	3.1	2.2	1.9
Osun	1.9	1.2	0.6	3.0	0.4	1.0	0.0	1.4
Oyo	6.6	2.3	2.7	2.4	1.6	3.7	2.2	2.8
Plateau	3.4	2.3	3.3	3.8	3.6	0.0	0.0	2.9
Rivers	0.6	5.3	3.5	2.2	5.2	2.6	2.2	3.8
Sokoto	1.9	3.1	2.3	0.9	2.0	0.5	8.9	2.3
Taraba	3.5	2.0	3.0	4.1	8.0	7.3	0.0	3.1
Yobe	8.9	4.0	5.3	7.2	8.8	15.2	4.4	5.9
Zamfara	1.3	1.8	4.3	3.0	3.2	0.5	2.2	2.6

Source: NASC (79).

The farmer–herder conflict dynamics from a security perspective undermine state authority and weaken community trust in governance structures. The Nigerian state’s limited capacity to enforce land rights, regulate grazing corridors, or implement comprehensive climate adaptation strategies has allowed localized disputes to fester into broader security crises. Internal displacement linked to farmer–herder conflicts has increased significantly, with affected households often unable to return to farming due to persistent insecurity (63). This erodes coping capacity and deepens poverty, creating fertile ground for further violence.

Resource conflicts also intersect with other forms of insecurity, including banditry, cattle rustling, and insurgency. In northwestern Nigeria, armed bandit groups exploit resource scarcity and weak governance to raid villages, displace farmers, and control access to farmland and markets (64). These dynamics illustrate how competition over natural resources not only triggers violence but also sustains cycles of insecurity that disrupt rural livelihoods and social cohesion.

### 4.3 Natural resource conflicts and insecurity implications on production, economic and human wellbeing in Nigeria

The consequences of natural disasters are clearly illustrated in Tables 7–11. Table 7 presents the proportion of communities affected by different natural disasters. A large share of respondents reported that all members of the community were affected, particularly for drought (59.8%) and crop diseases (37.2%). Flooding affects communities more variably, with 32.4% indicating that some parts of the community is affected, while 21.8% report total community impact. Livestock diseases also significantly affect large portions of communities, with 31.0% indicating that most of the community is impacted. This suggests that disasters often have widespread effects, extending beyond isolated households to entire communities.

**Table 7:** Proportion of the community affected by the disaster.

Proportion		Drought (102)	Flood (188)	Crop disease/pest (215)	Livestock disease (168)	Total (673)
10	A few of the community	1.0	14.9	8.8	16.1	11.1
25	Some of the community	13.7	32.4	18.1	29.8	24.4
50	Half of the community	8.8	13.8	11.6	7.7	10.8
75	Most of the community	16.7	17.0	24.2	31.0	22.7
100	All of the community	59.8	21.8	37.2	15.5	30.9
	Total	100.0	100.0	100.0	100.0	100.0

Source: NASC (79).

**Table 8:** Economic impact of natural resource disasters.

Disaster type	Loss due to production disruption (4,737)	Loss of revenue (3,650)	Other economic impact
Drought	10.5	9.7	10.8
Flood	42.7	42.0	39.4
Heavy rainfall	25.2	24.7	23.6
Extreme winds	15.5	16.5	19.2
Extreme temperatures (cold or heat)	3.1	2.9	1.5
Fire disaster	2.4	3.5	3.4
Other (specify)	0.7	0.8	2.0

Source: NASC (79).

**Table 9:** Physical impacts of natural resource disasters.

Disaster type	Crop losses (4,902)	Livestock losses (2,711)	Aquaculture losses (1,050)	Biomass losses (210)	Building damages or losses (4,028)	Other physical losses (44)
Drought	12.5	13.8	6.8	9.5	3.0	11.4
Flood	43.9	39.7	57.6	44.3	41.0	29.5
Heavy rainfall	24.9	26.0	22.2	21.9	29.2	25.0
Extreme winds	12.8	12.6	8.8	14.3	22.3	20.5
Extreme temperatures (cold or heat)	3.0	5.7	2.8	4.3	1.3	4.5
Fire disaster	2.3	1.9	1.0	4.8	2.8	4.5
Other (specify)	0.6	0.3	1.0	1.0	0.5	4.5

Source: NASC (79).

**Table 10:** Human impact of natural resource disasters lives and properties.

Disaster type	People killed	People rendered homeless (homes destroyed)	Hospitalized (1,838)	People displaced (2,054)	Other (483)
Drought	6.2	4.3	10.6	12.4	26.9
Flood	49.9	42.4	33.5	47.7	46.8
Heavy rainfall	22.5	28.7	27.6	24.5	10.1
Extreme winds	11.4	20.5	14.8	10.1	5.6
Extreme temperatures (cold or heat)	5.0	1.3	9.4	2.3	2.1
Fire disaster	3.4	2.2	3.2	2.1	7.0
Other (specify)	1.6	0.5	1.0	0.8	1.4

Source: NASC (79).

**Table 11:** Severity of physical and economic losses from disasters.

	Severity of physical losses				Severity of economic impact			
	Small losses	Significant losses	Almost total losses	Total	Small losses	Significant losses	Almost total losses	Total
Disaster type	6.1	4.7	3.6	5.0	6.6	4.7	4.0	5.1
Drought	15.7	21.0	29.0	20.3	16.2	21.4	28.8	20.8
Flood	14.7	12.5	8.4	12.8	14.8	12.4	8.6	12.6
Heavy rainfall	9.0	8.5	6.4	8.5	8.2	8.4	6.1	8.2
Extreme winds	3.1	1.5	0.5	1.8	2.9	1.4	0.6	1.6
Extreme temperatures (cold or heat)	1.2	1.4	2.0	1.4	1.1	1.4	1.6	1.4
Fire disaster	0.2	0.4	0.2	0.3	0.2	0.4	0.2	0.3
Other (specify)	50.0	50.0	50.0	50.0	50.0	50.0	50.0	50.0
Total	100.0	100.0	100.0	100.0	100.0	100.0	100.0	100.0

Source: NASC (79).

Table 8 presents the economic impacts of natural resource disasters. It shows that flooding is the most economically destructive hazard, contributing the highest of production disruption (42.75) and revenue loss (42.0%). This aligns with pa studies as floods have been identified as the costliest disaster type in Nigeria, crippling agricultural output and local economies (65).

Table 9 corroborates Table 8, with floods causing the greatest physical asset losses across crops (43.9%), livestock (39.7%), and aquaculture (57.6%). Heavy rainfall is the second most significant contributor, while extreme winds also cause considerable damage, especially to buildings (22.3%). Drought contributes moderately to crop and livestock losses but less infrastructural damage. The erosion of productive capital directly undermines livelihood security and reinforces poverty cycles, particularly in rural communities (66). Disruptions due to conflicts limit farmers’ access to land, inputs, labour and shortens the agricultural calendar, thus forcing farmers to either delay planting, reduce farm size, avoid distant plots that are perceived as unsafe or abandon fields during critical planting or harvesting periods. Livestock losses from cattle rustling and retaliatory attacks further reduce food supply and household income. George et al. (67) shows that areas affected by farmer–herder conflicts experience significant declines in crop yields, livestock holdings, farm incomes, loss of productive assets and erodes household coping capacity. These disruptions ripple outward: reduced food availability drives price inflation, heightens household vulnerability, and increases reliance on food imports or humanitarian aid.

Insecurity also disrupts food supply chains by restricting movement along rural roads, increasing transportation costs, and discouraging market participation. Traders are often reluctant to operate in high-risk areas, leading to reduced food availability and price volatility (68). This situation disproportionately affects poor households, who spend a larger share of their income on food and are less able to absorb price shocks. Investment in agriculture is also affected by insecurity, such that both smallholder farmers and larger agribusinesses are discouraged from adopting improved technologies or expanding production in conflict-prone areas. This contributes over time to structural food deficits and weakens national food security, particularly in staple crops such as maize, sorghum, and rice.

In some cases, natural resource conflicts lead to loss of lives and homes. Table 10 highlights the severe toll floods has on humans thereby resulting in the highest mortality (49.9%), displacement (47.7%) and homelessness (42.4%). Heavy rainfall is also associated with significant human impacts, particularly hospitalisation (27.6%) and homelessness (28.7%). Drought contributes less to fatalities but has notable indirect effects. The concentration of casualties and displacement from floods highlights failures in early warning systems, land-use planning, and post-disaster response, exposing deep-seated vulnerabilities (11). The consistently low impacts in the “Other (specify)” category across all impact tables suggest these events are either of lower magnitude, affect fewer people, or are less systematically recorded.

Table 11 presents the severity of both physical and economic losses resulting from natural disasters. Flooding recorded the highest proportion of severe losses, especially in the “almost total loss” category with 29.0% for physical losses and 28.8% for economic losses. Drought also contributes significantly to severe losses, while heavy rainfall and extreme winds contributed to moderate losses. Minor disasters such as fire and extreme temperatures contribute relatively little to overall severity.

This suggests that while multiple natural disasters occur, floods and droughts are the most devastating in terms of severity.

### 4.4 Climate change, natural resource conflict and insecurity nexus influence on food security in Nigeria

Table 12 presents the results of the Ordinary Least Square (OLS), Ordered Logit (Ologit), and Ordered Probit (Oprobit) estimations of factors influencing household food security status. The OLS model treats food security as a continuous outcome, while the Ologit and Oprobit models account for the ordinal nature of food security categories (severely insecure, mildly insecure, moderately insecure, and food secure). All the three models are statistically significant at the 1% level, indicating strong joint explanatory power of the included variables. The consistency in the signs and magnitude of coefficients across the models suggests robustness of the results.

**Table 12:** Estimate of the correlates of food security.

Explanatory variables	Oprobit	Ologit	OLS
Climate exposure	−0.193*** (0.057)	−0.316*** (0.093)	−0.205*** (0.058)
Insecurity exposure	−0.122* (0.057)	−0.196* (0.116)	−0.151** (0.073)
Av, annual temperature	0.007*** (0.003)	0.013*** (0.004)	0.010*** (0.003)
Av. Annual rainfall	0.000*** (0,000)	−0.001*** (0.000)	0.000*** (0.000)
Sex	−0.071 (0.074)	−0.122 (0.125)	−0.054 (0.071)
Household size	−0.021*** (0.007)	−0.036*** (0.012)	−0.019*** (0.007)
Age	0.002 (0.002)	0.004 (0.003)	0.002 (0.002)
Log of value of assets	0.175*** (0.019)	0.299*** (0.033)	0.156*** (0.017)
Educational level	−0.007 (0.006)	−0.011 (0.009)	−0.004 (0.006)
Credit access	−0.175 (0.131)	−0.280 (0.219)	−0.117 (0.126)
Sector	0.176*** (0.067)	0.302*** (0.112)	0.163** (0.066)
Access to extension services	−0.030 (0.049)	−0.062 (0.081)	−0.004 (0.049)
Log total household income	−0.011** (0.005)	−0.019** (0.008)	−0.014*** (0.005)
Farm size (hec)	0.001 (0.002)	0.002 (0.004)	0.001 (0.003)
North Central	0.350*** (0.093)	0.568*** (0.152)	0.346*** (0.098)
North East	0.201** (0.095)	0.338** (0.156)	0.187* (0.098)
North West	0.121 (0.102)	0.197 (0.169)	0.171* (0.102)
South East	−0.581*** (0.110)	−1.023*** (0.187)	−0.515*** (0.105)
South South	−0.445*** (0.114)	−0.786*** (0.195)	−0.398 (0,109)
Constant			−2.774*** (0.737)
cut1	3.541 (0,755)	6.156 (1,264)	
cut2	3.864 (0.756)	6.693 (1.267)	
cut3	3.899 (0.756)	6.752 (1.267)	
Number of obs	3,229	3,229	3,229
Wald chi2 (19)	396.20	380.13	20.57
Prob > chi2	0.000	0.000	0,000
Pseudo r-squared	0.069	0.071	0.1033

*p < 0.1, **p < 0.05; ***p < 0.01.

Source: GHS (2018/2019).

Climate disaster exposure is negative and statistically significant across all models, indicating that increased exposure to climate-related shocks such as flood, fire outbreaks, droughts, crops and livestock pests/diseases significantly reduces household food security. Specifically, a unit increase in climate disaster exposure decreases food security outcomes, reflecting its disruptive effects on agricultural production and livelihoods. This finding aligns with existing evidence that climate shocks reduce agricultural productivity and exacerbate food insecurity in vulnerable regions (69, 80). Similarly, insecurity exposure has a negative and significant effect on food security in the three models. This suggests that conflict-related disruptions such as restricted access to farmland, and displacement undermine household food access and availability. This result is consistent with studies showing that conflict reduces farm output, destroys assets, and weakens food systems (67).

Average annual temperature is positive and statistically significant across all models, implying that moderate increases in temperature may enhance agricultural productivity in certain contexts, possibly through longer growing seasons or improved crop conditions. However, we interpret this effect cautiously, as extreme temperature increases could have adverse effects on agricultural production. In contrast, rainfall shows mixed effects across models, suggesting that while rainfall is important, its impact on food security depends on distribution, timing, and intensity rather than total volume alone.

Household size is negative and statistically significant across all models, indicating that larger households are more likely to experience food insecurity due to increased consumption pressures. This supports the argument that rising household dependency ratios reduce per capita food availability, especially in resource-constrained settings. Asset ownership (log of value of assets) is positive and highly significant across all models, demonstrating that wealth accumulation enhances food security. Households with greater assets are better able to absorb shocks, invest in productive activities, and smooth consumption during periods of stress. This finding is consistent with previous studies emphasizing the role of assets in building resilience and improving welfare outcomes (70, 71).

In contrast, educational level and access to credit are not statistically significant, suggesting that these factors may not directly influence food security within the context of this study. This may reflect structural constraints such as limited access to quality education or ineffective credit systems, which reduce their potential impact on household welfare. Similarly, access to extension services does not show a significant effect, possibly due to inefficiencies in service delivery or limited coverage. Likewise, total household income (log) shows a negative and significant relationship with food security across models. This counterintuitive result may reflect income instability, unequal distribution, or the possibility that higher-income households face greater exposure to market-related risks. It may also indicate that income alone does not guarantee food security without stable access to food and effective resource allocation.

Regional and sector (location) effects are also pronounced. Sector is positive and statistically significant across all models thus suggesting that households in the rural areas are more food secure that their urban counterparts. Similarly, households in the North Central and North East regions show positive and significant coefficients, suggesting relatively better food security outcomes compared to the reference category. In contrast, households in the South East and South South regions exhibit negative and significant coefficients, indicating higher levels of food insecurity. These differences reflect spatial inequalities in access to resources, infrastructure, and livelihood opportunities, consistent with earlier findings that location plays a critical role in shaping food security outcomes (70, 72).

Overall, the results demonstrate that climate change and insecurity are key drivers of food insecurity in Nigeria, operating through multiple pathways including production disruption, asset loss, and market instability. At the same time, household assets play important protective roles. These findings underscore the need for integrated interventions that address both environmental and conflict-related drivers of food insecurity while strengthening household resilience.

#### 4.4.1 Robustness check and model diagnostics

This section presents the robustness checks based on the OLS estimation in Table 12 and the multicollinearity test reported in Table 13. We examined the presence of multicollinearity among the explanatory variables using the Variance Inflation Factor (VIF). The results presented in Table 13 indicate that all VIF values are well below the commonly accepted threshold of 0.5, with a mean VIF of 1.76. This confirms that there is no serious multicollinearity problem among the regressors, and the estimated coefficients are not biased due to high correlation among explanatory variables. The robustness of the model is further supported by the OLS results presented in Table 11, which show that the model is statistically significant at the 1% level, indicating strong explanatory power of the included variables in explaining variations in household food security. The consistency of coefficient signs and significance levels across the OLS, Ologit, and Oprobit models further strengthens confidence in the reliability of the results.

**Table 13:** VIF post estimation test for multicollinearity among the covariates included in the model.

Explanatory variables	VIF	1/VIF
Climate exposure	3.430	0.291
Insecurity exposure	3.340	0.299
Av, annual temperature	3.280	0.305
Av. Annual rainfall	3.020	0.331
Sex	2.790	0.358
Household size	2.350	0.425
Age	1.410	0.708
Log of value of assets	1.330	0.750
Educational level	1.320	0.760
Credit access	1.270	0.788
Sector	1.220	0.818
Access to extension services	1.170	0.852
Log total household income	1.150	0.866
Farm size (hec)	1.150	0.873
North Central	1.050	0.950
North East	1.050	0.952
North West	1.040	0.958
South East	1.020	0.981
South South	1.010	0.991
Mean VIF	1.760	

Source: GHS (2018/2019).

Consistent with the main findings, climate disaster exposure remains negative and statistically significant in the OLS model, confirming that increased exposure to climate shocks reduces household food security. This supports existing literature that highlights the adverse effects of climate variability on agricultural productivity and food access (69, 80). Similarly, insecurity exposure maintains a negative and significant relationship with food security, reinforcing the argument that conflict disrupts agricultural production, limits market access, and erodes household welfare (67, 68). These findings are consistent across model specifications, indicating that both climate shocks and insecurity are robust determinants of food insecurity in Nigeria.

Among household characteristics, household size remains negative and statistically significant, suggesting that larger households face increased pressure on available food resources. In contrast, asset ownership (log of value of assets) retains a strong positive and significant effect, indicating that wealth enhances resilience and improves food access. This aligns with asset-based welfare theories, which emphasize the role of asset accumulation in smoothing consumption and reducing vulnerability to shocks (70, 71). However, total household income (log) continues to show a negative and significant relationship with food security, even after robustness checks. This counterintuitive result may reflect income instability or unequal distribution within households, suggesting that income alone may not guarantee improved food access without effective allocation and stability.

Environmental variables also show consistent patterns. Average annual temperature remains positive and significant, while rainfall exhibits mixed effects, indicating that the relationship between climatic factors and food security is complex and context-specific. Regional effects observed in the OLS model are also consistent with the ordered models. Overall, the robustness checks confirm that the key determinants of food security climate disaster exposure, insecurity, household size, and asset ownership remain stable across model specifications. The absence of multicollinearity and the consistency of results across OLS, Ologit, and Oprobit models provide strong evidence of the reliability and validity of the estimated relationships.

### 4.5 Policy, governance, and adaptation responses to climate change, resource conflicts, and food security in Nigeria

The findings from the preceding sections highlight that climate change, natural resource conflicts, and insecurity jointly exert significant negative effects on food security in Nigeria. The high prevalence of severe food insecurity, combined with the strong negative effects of climate disaster exposure and insecurity observed in Table 11, underscores the need for integrated and well-coordinated policy responses. The intertwined nature of these challenges presents a complex policy problem that cannot be effectively addressed through isolated or reactive interventions.

Existing policy responses in Nigeria have largely been fragmented, ranging from anti-open grazing laws to food import subsidies, without adequately addressing the structural drivers of vulnerability such as resource scarcity, weak land governance, and climate variability. Several national frameworks—such as the National Climate Change Policy, the Agricultural Promotion Policy, and state-level land use regulations demonstrate growing recognition of climate risks and food security concerns. However, implementation has remained uneven and poorly coordinated, limiting their effectiveness in addressing the climate–conflict–food security nexus (62).

The prominence of land, livestock, and boundary-related conflicts (Tables 3, 4) suggests that governance of natural resources remains a critical gap. The anti–open grazing laws introduced in several states represent attempts to reduce farmer–herder conflicts. However, these policies often lack sufficient consultation with pastoral communities and are implemented without complementary investments in grazing reserves, water infrastructure, or alternative livelihood systems (73). As a result, they may unintentionally intensify competition over limited resources, thereby exacerbating conflict rather than resolving it.

Institutional fragmentation further constrains effective policy implementation. Overlapping mandates among federal, state, and local authorities weaken coordination in managing land, water, and climate adaptation initiatives. This challenge is compounded by climate-induced migration, as displaced populations move into new areas in search of viable livelihoods, increasing pressure on already scarce resources and heightening the risk of conflict (74).

The empirical results also show that climate disasters—particularly floods—have the most significant economic, physical, and human impacts (Tables 7–10), while also reducing food security outcomes (Table 11). This underscores the need for proactive disaster risk management strategies, including improved early warning systems, climate-resilient infrastructure, and effective post-disaster response mechanisms. Strengthening these systems would reduce vulnerability and limit the cascading effects of disasters on livelihoods and food systems (11).

Climate adaptation policies offer significant potential to simultaneously address resource conflicts and food insecurity if designed within a conflict-sensitive framework. Adaptation measures that increase resource availability—such as irrigation development, water harvesting systems, and rangeland restoration—can reduce competition over scarce resources and lower the likelihood of conflict. Access to such adaptive infrastructure can also enhance agricultural productivity and stabilize food supply, thereby improving household food security outcomes (75).

In addition, the positive role of asset ownership observed in Table 11 suggests that policies aimed at strengthening household resilience are critical. Promoting climate-smart agricultural practices such as the adoption of drought-tolerant crop varieties, improved storage technologies, and sustainable land management can enhance productivity and reduce vulnerability to climate shocks. These interventions not only improve food availability but also support income stability, thereby mitigating the adverse effects of both climate change and insecurity.

Furthermore, improving access to extension services and rural infrastructure can enhance the dissemination and adoption of these practices. Although extension access was not statistically significant in the model, strengthening institutional capacity and service delivery could improve its effectiveness in practice.

Overall, addressing the climate–conflict–food security nexus in Nigeria requires a holistic and coordinated policy approach. This includes strengthening natural resource governance, promoting inclusive and participatory policy design, investing in climate adaptation and rural infrastructure, and enhancing institutional coordination across levels of government. Without such integrated efforts, the persistence of climate shocks and resource-based conflicts will continue to undermine food security and sustainable development in Nigeria.

## 5 Conclusion

This study examined the nexus between climate change, natural resource conflicts, insecurity, and food security in Nigeria using nationally representative datasets and a combination of descriptive and econometric techniques. The findings provide strong empirical evidence that climate-related environmental changes and resource-based conflicts are deeply interconnected and jointly exert significant pressure on agricultural systems, economic performance, and household welfare.

First, the results show that climate variability manifested through droughts, floods, and other extreme events intensifies competition over scarce natural resources such as land and water. This, in turn, contributes to the persistence and escalation of conflicts, particularly those related to land use, livestock grazing, and boundary disputes. The dominance of these conflict types highlights the structural nature of resource-based tensions in Nigeria’s agrarian and pastoral systems.

Second, the study demonstrates that natural resource conflicts and associated insecurity have far-reaching implications for economic performance, agricultural productivity, and human wellbeing. Flooding emerges as the most economically and physically destructive hazard, significantly affecting production, income, and asset accumulation. Conflict-related disruptions further constrain access to farmland, reduce labour availability, and weaken market systems, thereby limiting agricultural output and increasing vulnerability among rural households.

Third, the econometric results confirm that the combined effects of climate shocks and insecurity significantly reduce household food security. Climate disaster exposure and insecurity both exhibit negative and statistically significant relationships with food security across all model specifications. In contrast, asset ownership improves resilience and enhance food security outcomes. These findings reinforce the argument that food insecurity in Nigeria is not only a function of poverty but also a consequence of environmental stress and conflict dynamics.

Overall, the study establishes that climate change acts as a stress multiplier, exacerbating existing vulnerabilities and intensifying resource competition, which ultimately undermines food security. Addressing food insecurity in Nigeria therefore requires a comprehensive approach that simultaneously tackles climate risks, resource governance challenges, and insecurity.

Based on the findings, the following policy recommendations are proposed:

First, there is a need for a coordinated and integrated policy approach that explicitly addresses the linkages between climate change, resource conflicts, and food security. Existing policies should be harmonized to ensure coherence across sectors such as agriculture, environment, and security. Strengthening institutional coordination among federal, state, and local governments will improve policy implementation and reduce fragmentation.

Second, there is a need to improve natural resource governance and land management systems. Given that land, livestock, and boundary disputes account for the majority of conflicts, reforms in land tenure systems and resource governance are critical. Policies should promote secure and equitable access to land, strengthen land administration systems, and encourage participatory conflict resolution mechanisms involving both farmers and pastoralists.

Third, there is a need to invest in programs that support non-farm employment opportunities, access to credit, and rural enterprise development can reduce dependence on climate-sensitive livelihoods and enhance resilience. Fourth, investment in climate adaptation and resilient agricultural systems is crucial. The significant negative impact of climate shocks on food security underscores the need for large-scale investment in climate adaptation. This includes the development of irrigation systems, water harvesting technologies, flood control infrastructure, and drought mitigation strategies. Promoting climate-smart agricultural practices such as drought-tolerant crop varieties, agroforestry, and improved storage systems can enhance productivity and resilience.

Fifth, there is a need to strengthen disaster risk management and early warning systems. Given the dominant role of floods in economic and human losses, improving disaster preparedness and response systems is essential. Investments in early warning systems, community-based disaster risk management, and climate information services can help households anticipate and respond to shocks more effectively. Strengthening post-disaster recovery programs will also reduce long-term impacts on livelihoods.

Despite the contributions made by this study, it has some limitations. First is with the use of secondary data which limits the ability to capture real-time dynamics and localized variations in climate shocks and conflicts. Second, the cross-sectional nature of the data restricts the analysis of causal relationships over time, particularly in understanding how climate change and conflict evolve and interact. Third, some variables such as insecurity and climate exposure are measured as binary indicators, which may not fully capture the intensity or frequency of these experiences. In addition, the analysis may not fully account for unobserved factors such as institutional quality, governance effectiveness, and social networks, which can influence both conflict dynamics and food security outcomes.

Future research should consider the use of panel data to better capture the dynamic relationships between climate change, conflict, and food security over time. Further studies could also incorporate other measures of conflict intensity and climate variability and explore the role of institutional and governance factors in mediating these relationships. Finally, future research should explore the effectiveness of specific policy interventions such as climate-smart agriculture programs, land reforms, and security initiatives in improving food security outcomes. This would provide more targeted evidence to guide policy design and implementation.

## Data Availability

The datasets presented in this study can be found in online repositories. The names of the repository/repositories and accession number(s) can be found at: [ACLED] [https://acleddata.com/country/nigeria] and National Bureau of Statistics (https://microdata.nigerianstat.gov.ng/index.php/catalog/80).
